# The Local Complement Activation on Vascular Bed of Patients with Systemic Sclerosis: A Hypothesis-Generating Study

**DOI:** 10.1371/journal.pone.0114856

**Published:** 2015-02-06

**Authors:** Cinzia Scambi, Sara Ugolini, T. Sakari Jokiranta, Lucia De Franceschi, Oscar Bortolami, Valentina La Verde, Patrizia Guarini, Paola Caramaschi, Viviana Ravagnani, Guido Martignoni, Chiara Colato, Serena Pedron, Fabio Benedetti, Marco Sorio, Fabio Poli, Domenico Biasi

**Affiliations:** 1 Department of Medicine, University of Verona, Verona, Italy; 2 Department of Bacteriology and Immunology, Haartman Institute and Research Programs Unit, Immunobiology, University of Helsinki, Helsinki, Finland; 3 Department of Pathology and Diagnostics, University of Verona, Verona, Italy; 4 Research Support Unit and Biostatistics, Verona University Hospital, Verona, Italy; University of Tennessee, UNITED STATES

## Abstract

**Objective:**

The role of complement system in the pathogenesis of systemic sclerosis (SSc) has been debated during the last decade but an evident implication in this disease has never been found. We carried out an explorative study on SSc patients to evaluate the expression of soluble and local C5b-9 complement complex and its relation with a complement regulator, the Membrane Cofactor Protein (MCP, CD46) on skin vascular bed as target distinctive of SSc disease. We also analyzed two polymorphic variants in the complement activation gene cluster involving the MCP region.

**Methods:**

C5b-9 plasma levels of SSc patients and healthy subjects were analyzed by ELISA assay. Archival skin biopsies of SSc patients and controls were subjected to immunofluorescence analysis to detect C5b-9 and MCP on vascular endothelial cells. The expression of MCP was validated by immunoblot analysis with specific antibody. Polymorphic variants in the MCP gene promoter were tested by a quantitative PCR technique-based allelic discrimination method.

**Results:**

Even though circulating levels of C5b-9 did not differ between SSc and controls, C5b-9 deposition was detected in skin biopsies of SSc patients but not in healthy subjects. MCP was significantly lower in skin vessels of SSc patients than in healthy controls and was associated with the over-expression of two polymorphic variants in the MCP gene promoter, which has been related to more aggressive phenotypes in other immune-mediated diseases.

**Conclusions:**

Our results firsty document the local complement activation with an abnormal expression of MCP in skin vessels of SSc patients, suggesting that a subset of SSc patients might be exposed to more severe organ complications and clinical evolution due to abnormal local complement activation.

## Introduction

Systemic sclerosis (SSc) is an autoimmune disease characterized by microvascular dysfunction, activation of the immune system and tissue fibrosis. Pathogenesis of SSc is complex and poorly understood and it has been suggested that a genetic predisposition might contribute to the development of the disease together with environmental agents, such as viruses or chemical agents, which could activate both cellular and humoral immunity [1, 2]. According to the current understanding, immune system leads to vascular injury with either release of pro-inflammatory cytokine or production of auto-antibodies that damage endothelial cells (ECs), resulting in promoted fibroblast proliferation [3–6].

So far the contribution of complement system to the pathogenesis of SSc has not been deeply investigated, most likely because in clinical practice the main plasma complement proteins (C3 and C4) are usually within the reference range. Nevertheless, hypocomplementemia has been described in SSc patients with more severe disease [7, 8], while high plasma levels of complement activation products have been correlated with clinical severity of SSc [9–12]. Recently, Batal et al. found that small vessel C4d score was higher in SSc patients with renal crisis compared with normotensive controls and that this score correlated with increased risk of unrecovered renal function [13]. Furthermore, Arason outlined a deficiency of complement-dependent prevention of immune precipitation in SSc [14] and Sprott et al. documented presence of the C5b-9 complex and C5a receptor in microvessels of SSc skin sections both in early and in late phases of the disease [15].

It is conceivable that activation of complement system in SSc might be due to immune complexes [16, 17], but inadequate protection of the EC surface might also be involved. In fact, ECs located at the interface between blood and tissues are natural targets of complement attack. The classical functions of complement, such as opsonization, recruitment of inflammatory cells, target cell lysis, immune complex clearance, and its capability to influence many other pathways, such as coagulation cascade and angiogenesis, seem to be pivotal for the integrity of ECs [18]. In normal conditions, complement attack is tightly regulated by fluid-phase and surface-bound regulatory proteins which allow adequate immune surveillance while ensuring protection of host cells [19]. In different vascular diseases, overtly activated or poorly controlled complement activation not only promotes EC damage and apoptosis, but also enhances the expression of vascular cell adhesion molecules and amplifies the local immune response [20].

Factor H (FH) is the main fluid-phase regulator of the alternative complement pathway (AP). It acts on C3, the central component of the complement cascade by accelerating decay of C3 convertase and acting as a cofactor of factor I (FI) in the inactivation of C3b. This plasma regulator also contributes to human tissue protection allowing complement activation only to foreign targets or altered self cells [21, 22]. In our previous study, we documented high FH levels in sera of SSc and Sclerodermatous Graft Versus Host Disease (ScGVHD) patients, but only in SSc subjects we found a defective capacity of FH to protect cellular surface from complement mediated damage in *in vitro* experiments [23].

On human ECs, other complement regulators participate in cell protection from activation of both AP and classical complement pathway (CP). The group of membrane-bound complement regulators include the membrane cofactor protein (MCP or CD46), which is a cofactor of FI in the proteolytic inactivation of C3b and C4b, and the decay accelerating factor (DAF or CD55), which accelerates the breakdown of C3- and C5-convertases [24–26]. Recently, Venneker et al. demonstrated an impaired expression of MCP and DAF in endothelium of the lesional and non-lesional skin of SSc patients and in the skin of patients with morphea, in comparison to healthy controls and subjects affected by other autoimmune diseases, suggesting that a defective endothelial protection might be mediated by reduced expression of the complement regulatory proteins [27, 28].

Since the mechanisms involved in SSc pathogenesis are still under investigation, we focused our attention on local complement activation and regulation, using skin biopsies as an observational window of the EC damage related to SSc.

## Materials and Methods

### Patient selection

The initial study population consisted of 71 SSc patients and 29 age- and sex-matched healthy volunteers (H). All SSc patients fulfilled the American College of Rheumatology criteria for the classification of SSc. Distinction between limited cutaneous SSc (lcSSc) and diffuse cutaneous SSc (dcSSc) was made according to the criteria of LeRoy et al [29]. Exclusion criteria for the SSc patients were co-morbidities associated with complement activation due to a potential confounding effect [30–32]. On this basis ten patients were excluded, so 25 subjects with dcSSc and 36 subjects with lcSSc were finally enrolled in the study.

All the enrolled patients underwent a detailed clinical examination and laboratory evaluation, including analysis of antinuclear antibodies by indirect immunofluorescence on HEp-2 cells, anti-ENA antibodies by an ELISA method, CRP determination and standard direct and indirect Coombs tests. Skin involvement was assessed modified Rodnan skin score (mRSS) [33] by evaluating dermal thickening in seventeen anatomic sites, using a score from 0 to 3 (where 0 indicates normal). Skin lesions were subdivided on the basis of skin score into mild (mRSS <14), moderate (mRSS between 15 and 29), high (mRSS between 30 and 39), and severe (mRSS ≥ 40) [34]. Moreover, all the patients underwent the following investigations: electrocardiogram, pulmonary function test with diffusing capacity for carbon monoxide adjusted to haemoglobin (DLCO), Doppler echocardiogram, and chest high-resolution computed tomography. Nailfold videocapillaroscopy (NVC) was assessed in 54 patients (25 with dcSSc and 29 with lcSSclcSSc) by a unique operator, who was unaware of the aims of the study. The microvascular alterations were classified into 3 different patterns: early, active, and late [35].

Archival skin punch biopsies of 8 patients with SSc (4 lesional SSc skins from wrists and 4 non-lesional SSc skins from backs), were processed and compared with those of 4 patients with ScGVHD (4 lesional GVHD skins from wrists, leg or forearm) and 8 healthy individuals (4 non-lesional H skins from wrists and 4 non-lesional H skins from backs). The institutional review board of the Verona Hospital approved the protocol (CE 1183/1570) and all patients and healthy controls provided written informed consent before participating in the study.

### Sample collection

Venous blood was drawn into 10 ml BD Vacutainer tubes and allowed to clot at room temperature for 1 hour. Venous blood (10 ml) was also collected in pre-cooled tubes containing 0.015 M sodium citrate and centrifuged immediately at 4°C. Serum or plasma was separated from cells by centrifugation at 3000 × *g* for 15 min at 4°C followed by an additional similar centrifugation in order to remove cellular debris. Serum and plasma samples were then aliquoted in 1.5 mL Eppendorf tubes and stored at -80°C until use.

### Enzyme-linked immunosorbent assay (ELISA)

Concentrations of FH in serum and SC5b-9 in plasma were assessed using the Human Complement Factor H ELISA kit (Hycult Biotech, Uden, The Netherlands) and the MicroVue C5a Plus EIA (QUIDEL), respectively, according to the manufacturers’ instructions. Serum samples were assayed after a 1:8000 dilution, while plasma samples were assayed using a 1:10 dilution.

Concentrations were calculated using standard curves generated with specific standards provided by the manufacturers. Optical density was measured by microtitre plate reader at 450 nm. Each sample was measured in duplicate.

### FH-dependent hemolysis assay

The hemolysis test was performed as previously described [36]. Briefly, 100 μl of each serum was diluted in 400 μl of alternative pathway activating buffer (AP buffer: 2.5 mM barbital, 1.5 mM sodium barbital, 100 mM NaCl, 7mM MgCl_2_, 10 mM EGTA pH 7.2–7.4). A duplicate of each sample was prepared in the same buffer plus 50 mM EDTA and was used as blank. 200 μl of sheep erythrocytes (1x10^8^ cells/ml in AP buffer) were added to both samples and blanks. The mixtures were incubated at 37°C under mixing. After 15 min, samples were transferred to 0°C and the reaction was stopped with 1 ml of stop solution (2.5 mM barbital, 1.5 mM sodium barbital, 144 mM NaCl, 2 mM EDTA, pH 7.2–7.4). A sample with 400 μl of AP buffer and 200 μl of sheep erythrocytes, incubated in the same conditions but stopped with 1 ml of stop solution plus 0.1% Triton X-100 was used as the “control of total lysis”.

The mixtures were centrifuged at 2600 × g for 15 min and hemolysis was determined by measuring the absorbance at 414 nm in the supernatants. The percentage of lysis in each sample was calculated as percentage of the absorbance of the sample divided by that of the “control of total lysis” (OD at 414 nm). Samples were considered positive when percentage of lysis was higher than 12.5%; spontaneous lysis of sheep RBC in the presence of normal human serum was 0.1–12.15%.

### Western blotting

Proteins were extracted from skin specimens by Tri-Reagent method (Sigma) and quantified using BCA assay (Pierce Company). Sixty micrograms of proteins were subjected to electrophoresis in SDS-(12%) polyacrylamide gel under reducing conditions and then blotted to a nitrocellulose membrane using the Mini Trans-Blot Cell (Bio-Rad). The membranes were incubated with blocking buffer (3% w/v skimmed milk in Tris Buffered Saline and 0.1% v/v Tween-20) and then probed with 1:5,000 primary rabbit anti-human MCP antibody (Abcam), under shaking for 1 hour at 22°C. After the washing and the incubation with 1:15,000 horseradish peroxidase-conjugated anti-rabbit antibody (Abcam) for 1 hour at 22°C, the immunocomplexes were detected by chemiluminescence using the ECL Plus Western Blotting Detection Reagents (GE Healthcare Life Sciences). We developed the images on Kodak BioMax XAR Films in the darkroom, adjusting the exposure time depending on the intensity of the protein bands. Blots were stripped by adding a Stripping Acid Solution (50 mM glycine, 1% w/v SDS, 1% w/v Tween-20; pH 2.2 with HCl), shaking for 40 min at 37°C and reincubated with mouse monoclonal anti-human ß-tubulin antibodies (1:1,000 dilution; Abcam) to confirm the equal sample loading of the gels and the efficiency in electrophoretic transfer. Densitometric analysis of the bands was performed using Quantity One software (Biorad).

### C5b-9 and MCP immunofluorescence assay

Paraffin-embedded tissue blocks were cut into 2–3 μm sections and mounted on adhesive microscope glass slides. After deparaffinization and rehydration the antigen retrieval was performed in pre-warmed citrate buffer (pH 6 temp. 95°C) for 30 minutes. Sections were cooled to room temperature and incubated with a protein blocking serum-free solution for 15 minutes at 22°C to block non-specific binding.

For immunofluorescence staining, sections were separately incubated with three different antibodies: a monoclonal mouse anti-human CD31 antibody (Dako, JC70A, 1:50 dilution; a marker for vascular endothelium), a monoclonal rabbit anti-human CD46 antibody (Abcam, ab108307, 1:500 dilution), and a monoclonal murine anti-human SC5b-9 antibody (QUIDEL, A239, 1:250 dilution). Slides were incubated 30 minutes at pH 6 with the corresponding Alexa 546-conjugated antibody (anti-mouse or anti-rabbit; INVITROGEN Molecular Probe, diluted 1:1000). Reduction of the autofluorescence background was obtained by the incubation with Sudan Black B 0.1% (Sigma-Aldrich). Nuclei were stained with Prolong Gold antifade reagent with DAPI (INVITROGEN Molecular Probe). Slides were analysed by a Olympus BX61 microscope.

### Genetic analysis

DNA was extracted from the blood buffy coat by automated Blood DNA purification kit on the Maxwell 16 instrument (Promega), according to the manufacturer’s instructions. DNA preparates were stored at -80°C until the analyses were performed.

DNA samples of six SSc patients, which resulted positive to the hemolysis test, were sent to Secugen Diagnostic (Madrid, Spain) for the genetic analyses. Exons and promoter regions of genes for FH, FI and MCP were sequenced and compared to the published sequences in Ensemble, NCBI, and aHUS databases. Genotypes and haplotypes for common polymorphisms (SNPs) in these genes were also analyzed. The two polymorphic variants of *MCP* promoter region (-366A>G, rs2796268 and -652A>G, rs2796267), of those identified thereby, were assessed with TaqMan allelic discrimination assays designed on demand (Applied Biosystems, Foster City, CA, USA) on a 7500 Real Time PCR instrument (Applied Biosystems).

### Statistical analysis

Differences in FH concentrations was evaluated by Kruskal Wallis test, as this variable presented a highly skewed distribution according to Skewness—Kurtosis test. The Mann Witney test was performed, adjusting for multiple comparisons by Bonferroni correction, if the Kruskal Wallis test result was significant. The same analyses were performed for C5b-9 and CD46 vessel ratios.

The differences in categorical variables, including allele frequencies of each SNP, were evaluated by the Fisher’s exact test. Significance was set at *p*<0.05.

## Results

### Demographic and clinical data of SSc patients

The main clinical characteristics of the SSc patients studied are listed in Table 1. Twenty SSc patients presented anti-Scl70 antibodies, thirty-one subjects were positive for anti-centromere antibodies, and ten patients presented antinuclear antibodies. The mRSS score documented the presence of mild disease in 73.8% and of moderate disease in 24.6% of the cases. One patient only had high skin involvement. Evidence of interstitial lung disease was found in seventeen patients (27.9%).

**Table 1:** Clinical data of SSc patients.

**Age** (years)		61.6±13.1
**Sex** §	men	11 (18.0%)
	women	50 (82.0%)
Disease pattern§	dcSSc	25 (41.0%)
	lcSSc	36 (59.0%)
Autoantibody pattern§	Anti-Scl70	20 (32.8%)
	ACA	31 (50.8%)
	ANA	10 (16.4%)
mRSS§	mild	45 (73.8%)
	moderate	15 (24.6%)
	severe	1 (1.6%)
NCV pattern§	early	12 (19.7%)
	active	19 (31.1%)
	late	23 (37.7%)
	missing	7 (11.5%)
Pulmonary fibrosis§	present	17 (27.9%)
	absent	44 (72.1%)

Anti-Scl70 = anti-Scl70 antibodies; ACA = anticentromere antibodies; ANA = antinuclear antibodies; mRSS = modified Rodnan Skin Score; NVC = nailfold videocapillaroscopy.

§ values expressed as absolute number and percentages.

At videocapillaroscopic analysis, twelve subjects showed early microvascular alteration pattern, while nineteen and twenty-three patients had respectively active and late nailfold microvascular damage (Table 1).

### In SSc, FH levels are increased in presence of normal soluble C5b-9 complement complex

Previously, we have reported that FH is increased in serum of the SSc patients [23]. Here, we evaluated C5b-9 plasma concentrations as an index of systemic complement activation. We found that C5b-9 plasma concentrations were similar in the SSc patients and healthy controls (dcSSc 134 ng/ml, IQR 93–203; lcSSc141 ng/ml, IQR 89–202; H 124 ng/ml, IQR 82–159; p = 0.49); whereas, the serum levels of FH were higher in SSc patients with both diffuse and limited subsets (dcSSc patients 126 μg/ml, IQR 114–150, p = 0.0025; lcSSc patients 124 μg/ml, IQR 108–152; p = 0.0054), compared to healthy controls (108 μg/ml, IQR 93–120 μg/ml), according to the Bonferroni correction.

No correlation was observed between serum FH concentrations and CRP levels in the samples from the SSc patients (p = 0.93; data not shown), although CRP had been reported to correlate with FH in patients with age-related macular degeneration (AMD) [37].

### The function of FH is impaired in SSc patients

The presence of normal values of C5b-9 in the peripheral circulation of SSc patients with increased levels of FH, made us to consider local activation of complement. To study the regulatory activity of FH on cell surfaces, we carried out a hemolysis assay in the presence of Mg-EGTA, which selectively blocks CP and lectin pathway of complement (LP). As shown in Table 2, the FH-dependent hemolysis test revealed that 40% and 16% samples of the dcSSc and lcSSc groups were positive, respectively, whereas healthy controls were all negative (p<0.001. In addition, we found that the serum hemolytic activity was higher in SSc patients with high levels of FH (p = 0.012) (Table 2).

**Table 2:** Serum hemolytic activity in healthy subjects and SSc patients.

	Healthy controls		SSc patients	
Hemolysis test	Negative [n = 29]	Positive [n = 0]	Negative [n = 45]	Positive [n = 16]
FH serum level, mean (IQR) [μg/ml]	108 (93–120)	-	122 (103–139)	151 (122–186)

To exclude possible additional factors that might interfere with the hemolysis test (i.e. antibodies against sheep red cells), we carried out the direct and the indirect Coombs tests, which resulted negative in all the SSc patients studied (data not shown).

### Serum hemolytic activity in SSc is mediated by the alternative and classical complement pathways

**Fig 1:**
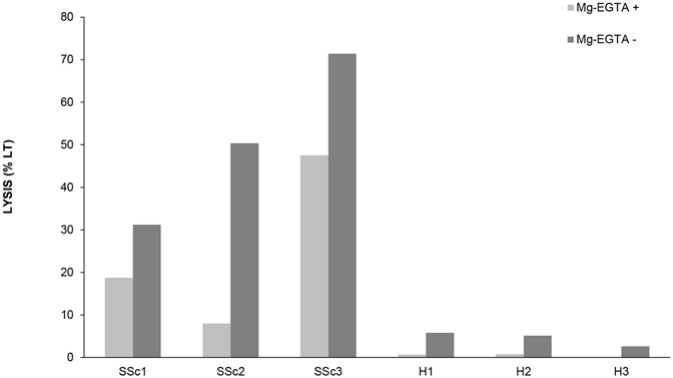
Serum hemolytic activity is increased in SSc patients. Sera from three different SSc patients (SSc1-SSc3) and three different healthy subjects (H1–H3) were tested both in the presence and absence of Mg-EGTA. Mg-EGTA was used to block the classical and lectin pathways of complement. Data are expressed as percentage of full RBC lysis.

Since the local complement activation could be mediated by any of the three complement pathways, we evaluated the serum hemolytic activity of SSc patients both in the presence and absence of Mg-EGTA. Interestingly, the hemolysis in the absence of Mg-EGTA was higher than in presence of Mg-EGTA (Fig. 1), suggesting a possible concert action of both AP and CP/LP in the complement dysregulation on the cell surface.

### The endothelium of SSc patients is not protected from activated complement

**Fig 2:**
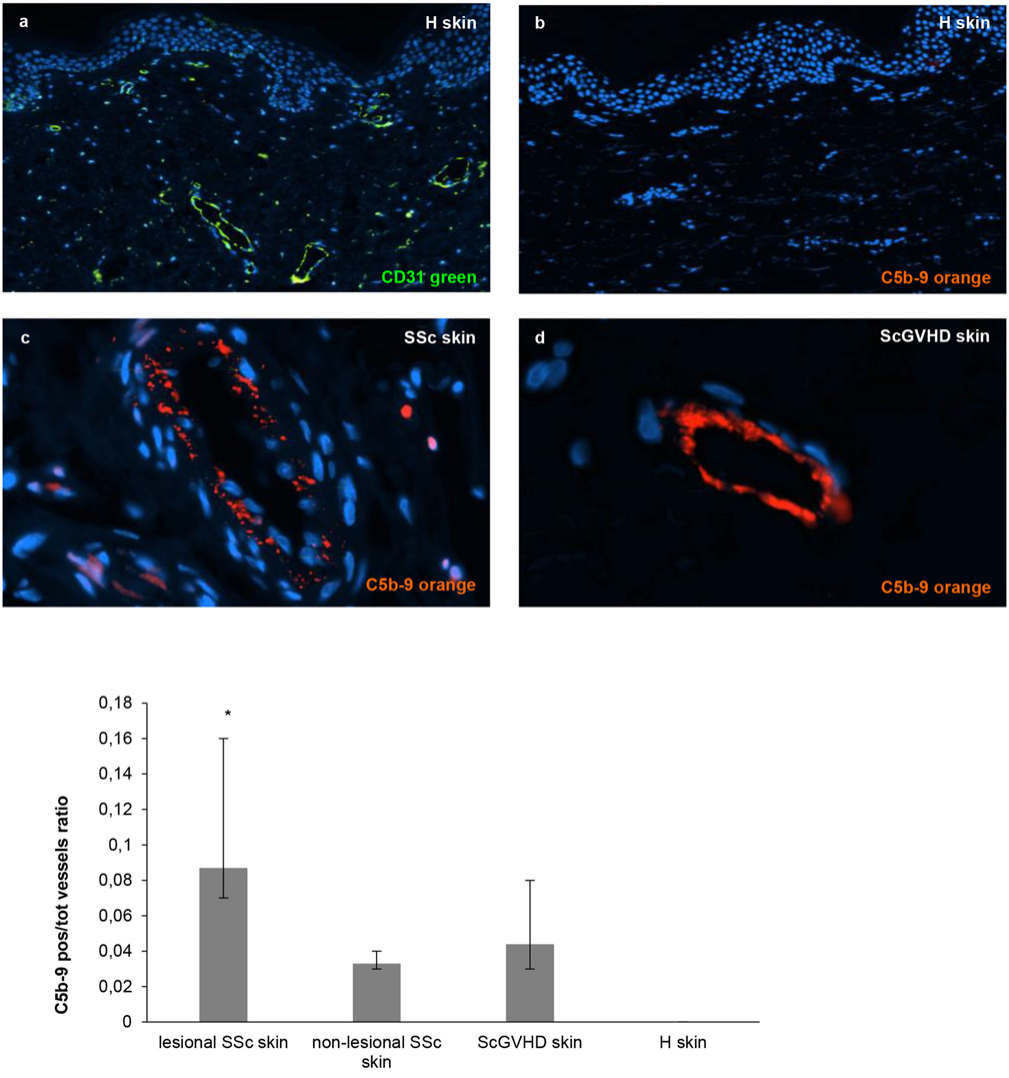
Detection of the complement membrane attack complex (or C5b-9) on skin endothelial cells of SSc patients. Skin biopsies from 8 SSc patients (4 lesional SSc skin and 4 non-lesional SSc skin), 8 healthy subjects and 4 patients with ScGVHD were immunostained for C5b-9 specific antibody (orange). Anti-CD31 antibody (green) was used as marker of endothelial cells and DAPI to stain the nuclei of the cells. Data of the analysed fields (*n* = 20 for each slide) are expressed as a ratio between C5b-9 positive vessels and total number of vessels.

Considering that the vascular endothelial bed is involved in the early phase of SSc, we investigated whether the ECs of SSc patients might be exposed to complement mediated damage, as previously suggested by Sprott [15]. To study this we compared archival skin biopsies of 8 SSc patients showing positive hemolysis test to those of 4 subjects with ScGVHD and 8 normal individuals. As shown in Fig. 2, C5b-9 was detected in vessels of SSc and ScGVHD skin, while microvasculature of healthy subjects resulted completely negative (median proportion of positive vessels—H: 0.000; lesional SSc: 0.087; non-lesional SSc: 0.033; ScGVHD: 0.044). Pairwise comparisons after Kruskal Wallis test documented a significant difference between the samples from healthy skin and those from lesional SSc (p = 0.0002; Bonferroni-adjusted level for significance = 0.0042) (Fig. 2).

**Fig 3:**
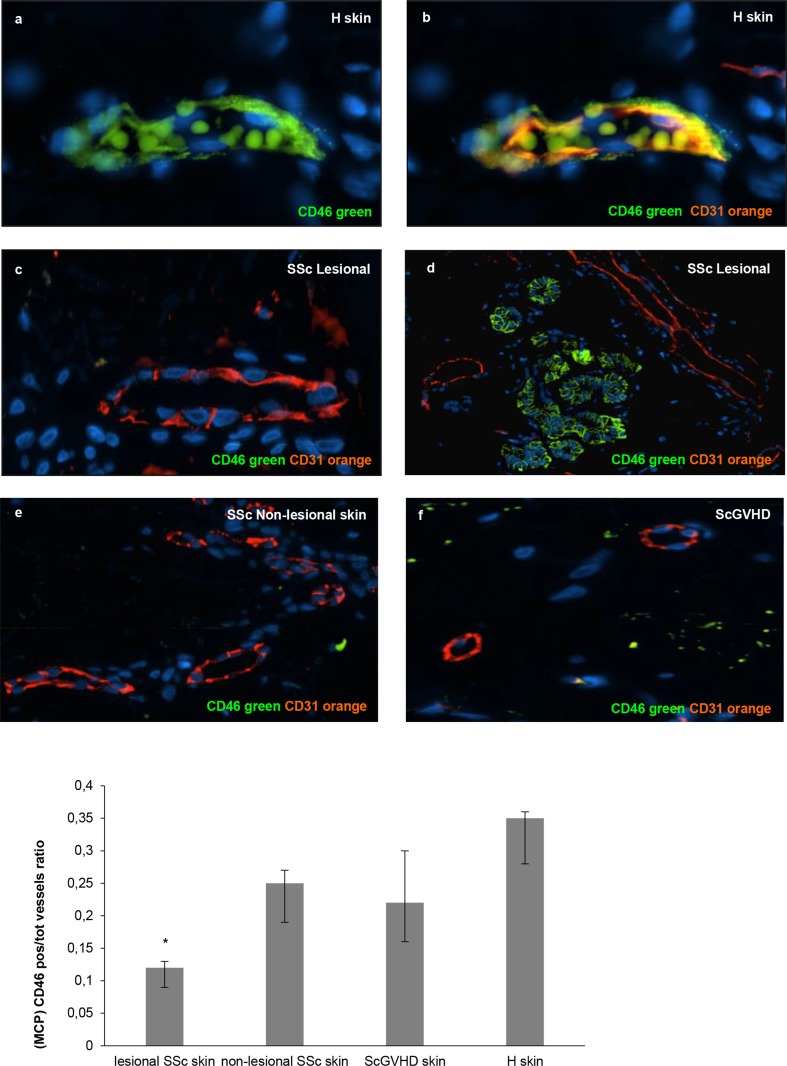
The local complement regulator MCP (CD46) is reduced on vascular endothelial cells of SSc skin biopsies. Skin sections from 8 SSc patients (4 lesional SSc skin and 4 non-lesional SSc skin), 12 controls (8 healthy subjects and 4 ScGVHD patients) were immunostained with anti-CD46 (green) specific antibody. Anti-CD31 antibody (orange) was used as a marker of endothelial cells. Data of the analysed fields (*n* = 20 for each slide) are expressed as ratio between MCP (CD46) positive vessels and total number of vessels.

**Fig 4:**
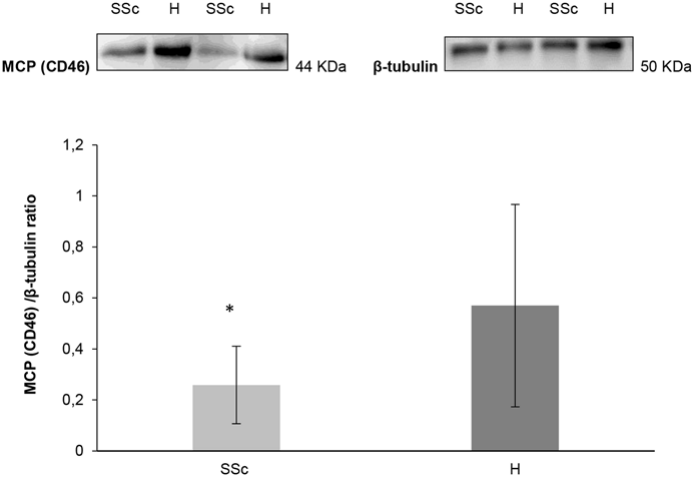
Immunoblot analysis of MCP (CD46) of skin biopsies from healthy controls and SSc patients. Protein extract from skin biopsies of 8 SSc patients (4 lesional SSc skin and 4 non-lesional SSc skin) and 8 healthy subjects were analyzed by immunoblot using a specific antibody. Results of a representative experiment of four are shown. Data are expressed as an optical density ratio of MCP (CD46) and β-tubulin.

This finding supported the local C5b-9 deposition around blood vessels of SSc skin. Thus, we looked for the expression of MCP, which is normally expressed on ECs and acts as a local complement regulator. The immunofluorescence staining revealed significantly reduced amount of MCP in the endothelium of lesional SSc skin than in the healthy controls (median proportion of positive vessels—H: 0.35; lesional SSc: 0.12; non-lesional SSc: 0.25; ScGVHD: 0.22; p value of pairwise comparisons H *vs* lesional SSc = 0.0004; Bonferroni-adjusted level for significance = 0.0042) (Fig. 3). Immunoblot analysis provided additional evidence of low expression of MCP in SSc skin sections (median proportion of CD46 H: 0.570; SSc: 0.258; p = 0.03 *vs* H) (Fig. 4).

### SSc population has higher prevalence of SNPs polymorphic variants in MCP promoter region

The sequencing analysis of FH, FI and MCP genes revealed one heterozygous mutation in FI gene (c.1534+5G>T) in one of the six analyzed patients (patient n. 1), while no mutations were found in FH and MCP genes. Homozygosis for non-prevailing SNPs of FH gene were found in three subjects (patients n. 2, n. 3 and n. 6), instead homozygosis for uncommon SNPs of MCP gene were detected in four patients (patient n. 1, n. 2, n. 3 and n. 4), as shown in S1 Table. Only patient n. 5 did not present mutations or non-prevailing SNPs in the analyzed genes.

Since expression of MCP was different in SSc and healthy skin, we next analyzed prevalence of the two allelic variants in the promoter region of the *MCP* gene (-366A>G and -652A>G SNPs) in all the SSc patients and healthy subjects. These polymorphisms have been reported to correlate with the disease severity of the atypical hemolytic uremic syndrome (aHUS) [38].

We found differences in the distribution of both the MCP polymorphisms between the SSc patients and healthy controls despite the small size of our sample. In particular, in SSc the allele frequency reached the statistical significance for -366G (0.42 *vs* 0.24; p = 0.021), whereas for -652G the analysis were close to significance (0.42 *vs* 0.27; p = 0.071) compared to the controls (Table 3).

**Table 3:** Allele frequencies of two SNPs in MCP gene promoter.

SNPs	NCBI id	SSc patients					Healthy controls					*P*-values
		Genotypes			Allele frequencies		Genotypes			Allele frequencies		
		1/1	1/2	2/2	1	2	1/1	1/2	2/2	1	2	
-366A>G	rs2796268	23	36	12	0.58	0.42	16	9	2	0.76	0.24	0.021
-652A>G	rs2796267	22	38	11	0.58	0.42	15	9	3	0.72	0.28	0.071

The *P-*value is the result of a two-sided Fisher exact test for the comparison of the allelic frequencies of each SNP between the SSc and control groups.

## Discussion

Here, we propose the endothelium-bound membrane attack complex of complement (MAC or C5b-9) as a promising marker of active vascular damage in SSc despite its normal plasma levels. Previous studies in other autoimmune diseases have found similar discrepancy between plasma levels of complement activation products and local complement activation. In *SLE*, *the* activation of complement system has been reported in different organs, such as lung and kidney, without changes in serum levels of C3 and C4 [39]. In rheumatoid arthritis the presence of complement activation fragments in joint fluid and the deposition of C5b-9 in synovial tissue are common findings, too, although the plasma level of C5b-9 may not be altered [40].

In this study, we used erythrocytes as a model of cellular surface sharing common characteristics with other cell types. The serum hemolytic activity of SSc patients does not, however, represent well complement activation and regulation on endothelium since red blood cells are devoid of MCP. Thus, we studied SSc skin biopsies as an observational window of endothelial damage in SSc. We found that the complement system is locally activated, as documented by the abnormal deposition of MAC on the endothelium and, concordant with this, we found reduced MCP expression on vascular endothelial surface. This finding is in agreement with that previously reported by Venneker on SSc skin [27]. Based on these results we propose that the lack of the fine local regulation of complement activation on vascular endothelium might promote complement activation leading to sublethal MAC depositions which are known to cause EC apoptosis. This could explain at least partially the initiation or propagation of tissue fibrosis in SSc.

In other disorders characterized by abnormal complement regulation (i.e. aHUS and AMD), it has been shown that mutations or uncommon SNPs of complement regulatory proteins, such as FH and MCP, generate a state of haploinsufficiency unable to prevent complement mediated tissue damage [41–44]. As some SNPs are organized in specific haplotype blocks within the regulator of complement activation gene cluster in human chromosome 1q32, we examined two SNPs in the MCP promoter region (-366 A>G, -652 A>G) showing a strong linkage disequilibrium in the region of the MCP gene. It is of interest to note that the polymorphic variants of these two SNPs have been related to a 25% lower transcriptional activity of the gene promoter and have been linked to enhanced severity of aHUS disease [38]. In our SSc patients, the minor variants of the two SNPs were more usual than in healthy controls, suggesting a possible role of these SNPs in the severity of SSc disease. It remains to be studied if the SNP -366A>G in the MCP gene could be used as a predictive marker for more severe or progressive disease.

Beside the clinical similarities between SSc and ScGVHD, we did not observe the prevalence of the same polymorphic variants in the promoter region of MCP gene in ScGVHD patients studied (data not shown). Moreover, the observation that in skin biopsies of ScGVHD patients the local amount of C5b-9 was increased without significant differences in the MCP expression, supports the hypothesis of additional mechanisms involved in complement activation on skin endothelium in ScGVHD. The important role of complement system in transplant rejection reactions is supported by the encouraging results with the anti-C5 monoclonal antibody (Eculizumab) that blocks the activation of the terminal complement cascade and formation of MAC [45, 46].

**Fig 5:**
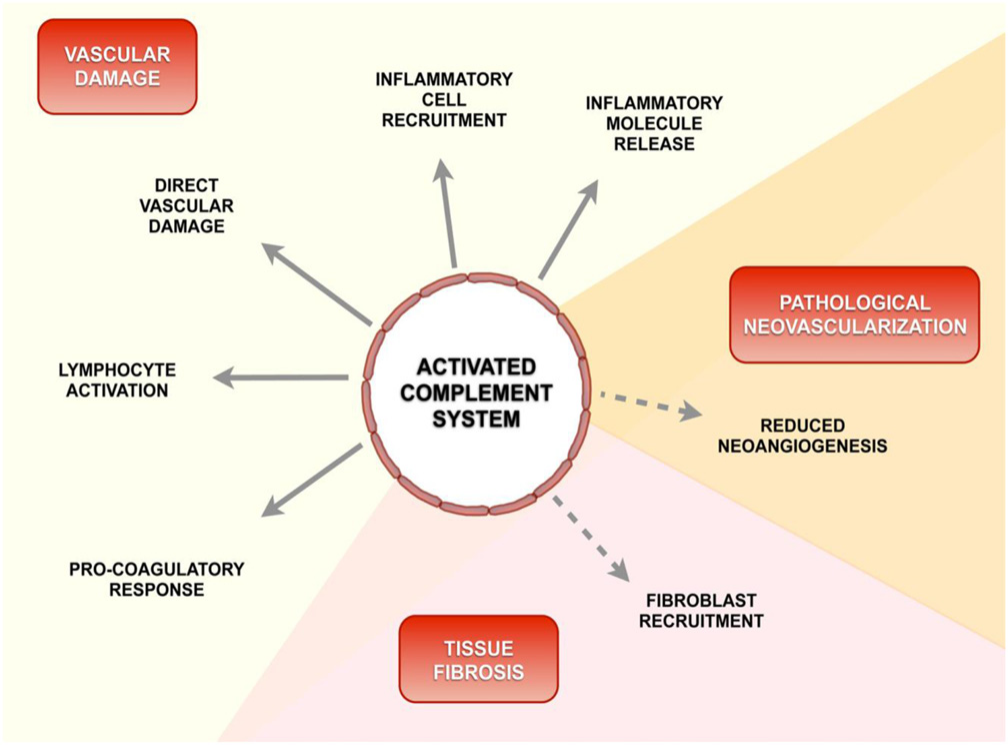
Schematic diagram of a possible role of activated complement in the pathogenesis of SSc. Beside its conventional role in the innate immunity, recent evidence suggests that complement system modulates the acquired immunity and regulates the coagulation cascade activation. Locally, activated complement products reduce neoangiogenesis and promote tissue fibrosis.

We propose that several mechanisms known to be involved in SSc pathogenesis might be affected by locally activated complement caused by impaired fine-tuning of this powerful innate immune defense (Fig. 5). In fact, different pathways are likely to contribute to vascular dysfunction processes in SSc, such as direct vascular damage, pro-inflammatory response and coagulation cascade activation. Studies in different diseases have shown functional connections between activated complement molecules and these pathways [18, 47–49].

In aHUS dysregulated complement activation is clearly causing the endothelial cell injury, hemolysis and microvascular thrombosis. In addition, in models of multifactorial disease (e.g. antiphospholipid syndrome) a partial or complete loss of function of complement regulators might play a relevant role in the pathogenesis, contributing to more severe organ damages and clinical complications. The revision of the literature on these disorders confirms that pharmacological treatment with Eculizumab might ameliorate clinical manifestations in severe cases [50, 51].

Future studies need to be carried out to better characterize the role of complement system on vascular damage in SSc and to verify in a large number of SSc patients the possible beneficial effects of a pharmacological treatment with inhibitors of complement system.

## Supporting Information

S1 Table SNPs of FH and MCP genes. (DOCX)
