# Supplementary material for: The Local Complement Activation on Vascular Bed of Patients with Systemic Sclerosis: A Hypothesis-Generating Study
Source: PLoS One. 2015 Feb 6;10(2):e0114856. doi: 10.1371/journal.pone.0114856 (PMC4319765; doi:10.1371/journal.pone.0114856)
Supplement: S1 Table — (DOCX) [file pone.0114856.s001.docx]

**Table S1.** **SNPs of FH and MCP genes.**

| **SNPs** | NCBI id | **SSc patients** | | | | | |
| --- | --- | --- | --- | --- | --- | --- | --- |
| [Allele1>Allele2] |  | **1** | **2** | **3** | **4** | **5** | **6** |
| FH c.184G>A | rs800292 | G/G | G/A | G/G | G/A | G/A | A/A |
| FH c.1204T>C | rs1061170 | T/C | T/T | C/C | T/T | T/T | T/T |
| FH c.2016A>G | rs3753396 | A/A | A/G | A/A | A/A | A/G | A/A |
| FH c.2808G>T | rs1065489 | G/G | G/T | G/G | G/G | G/T | G/G |
| FH c.257C>T | rs3753394 | C/T | C/T | C/C | C/T | C/T | C/C |
| FH c.233C>T | rs74842824 | C/C | C/C | C/C | C/C | C/C | C/C |
| FH 427+57_58insA | rs35836460 | - | WT/insA | - | - | - | - |
| MCP -652A>G | rs2796267 | G/G | G/G | G/G | G/G | A/G | A/G |
| MCP -366A>G | rs2796268 | A/G | G/G | G/G | G/G | A/G | A/G |
| MCP c.989-78G>A | rs1962149 | G/A | A/A | A/A | A/A | G/A | G/A |
| MCP *897T>C | rs7144 | T/C | C/C | C/C | C/C | T/C | T/C |

Allele 1 is the common allele in healthy control population.
